# Design and Application of a Radiofrequency Spectrophotometry Sensor for Measuring Esophageal Liquid Flow to Detect Gastroesophageal Reflux

**DOI:** 10.3390/s25113533

**Published:** 2025-06-04

**Authors:** Pedro J. Fito, Ricardo J. Colom, Rafael Gadea-Girones, Jose M. Monzo, Angel Tebar-Ruiz, F. Javier Puertas, Marta Castro-Giraldez

**Affiliations:** 1 Instituto Universitario de Ingeniería de Alimentos FoodUPV, Universitat Politècnica de València, Camino de Vera s/n, 46022 Valencia, Spain; pedfisu@tal.upv.es; 2 Instituto de Instrumentación para Imagen Molecular I3M, Universitat Politècnica de València, Camino de Vera s/n, 46022 Valencia, Spain; rcolom@upv.es (R.J.C.); rgadea@eln.upv.es (R.G.-G.); jmonfer@upvnet.upv.es (J.M.M.); atebar@upvnet.upv.es (A.T.-R.); 3 Neurophysiology and Sleep Unit, La Ribera University Hospital-FISABIO, 46600 Alzira, Spain; fj.puertas@ucv.es; 4 Faculty of Medicine and Health Sciences, Catholic University of Valencia “San Vicente Mártir”, 46001 Valencia, Spain

**Keywords:** radiofrequency spectroscopy, GERD, reflux, bioimpedance

## Abstract

**Highlights:**

**What are the main findings?**

- A radiofrequency spectrophotometry sensor was developed for the non-invasive de-tect GERD by measuring esophageal liquid flow and ionicity;
- It identified beta dispersion relaxation as a biomarker for esophageal tissue damage and alpha dispersion conductivity as an indicator of reflux, showing high sensitivity.

**What is the implication of the main finding?**

- The sensor offers a patient-friendly, real-time alternative to endoscopy and pH moni-toring, eliminating associated discomfort;
- This innovation enables continuous GERD monitoring, enhancing diagnostic accu-racy and supporting long-term tracking in clinical and ambulatory care.

**Abstract:**

Gastroesophageal reflux disease (GERD) is a widespread condition that requires reliable and non-invasive diagnostic methods to minimize patient discomfort. This study presents a radiofrequency spectrophotometry sensor specifically designed to detect esophageal liquid flow and ionicity in real time without disrupting the patient’s daily life. The sensor operates by measuring dielectric properties and ionic conductivity through the thoracic plexus, eliminating the need for invasive probes or prolonged monitoring. A study conducted on 49 participants demonstrated the sensor’s ability to differentiate between various liquid media and identify beta dispersion relaxation as a biomarker for esophageal tissue damage, a key indicator of GERD progression. Additionally, alpha dispersion conductivity effectively distinguished reflux episodes, proving the sensor’s high sensitivity. Unlike traditional diagnostic techniques such as endoscopy or pH monitoring, this radiofrequency spectrophotometry sensor enables continuous, real-time reflux detection, allowing patients to maintain a normal lifestyle during assessment. The results validate its potential as an innovative alternative for GERD diagnosis and monitoring, with future research focused on clinical validation, optimization, and integration into long-term patient monitoring systems.

## 1. Introduction

Gastroesophageal reflux disease (GERD) is a chronic condition characterized by the abnormal flow of stomach contents, including acid, into the esophagus. GERD can lead to a wide variety of symptoms, from mild heartburn to severe complications. According to various studies, GERD affects approximately 10–30% of the global population [1], with the prevalence being particularly high in Western countries. This condition is not only uncomfortable but also associated with long-term health risks such as esophagitis, Barrett’s esophagus, and esophageal adenocarcinoma [2].

The primary cause of GERD is dysfunction of the lower esophageal sphincter (LES), the valve that separates the stomach from the esophagus [3]. Under normal conditions, the LES prevents stomach contents from flowing back into the esophagus. However, in individuals with GERD, this sphincter is often weak or relaxes inappropriately [2]. While GERD is often diagnosed based on symptoms alone, particularly in individuals experiencing classic symptoms such as heartburn and regurgitation, several diagnostic tests are available to confirm the condition and assess potential complications. These tests are especially useful for patients with atypical symptoms, treatment-resistant GERD, or suspected complications such as Barrett’s esophagus or esophageal cancer [4]. However, many of these techniques are invasive, uncomfortable, and may be contraindicated in patients with certain digestive disorders.

These techniques can be classified as follows [3]. The first technique is the upper endoscopy esophagogastroduodenoscopy, a commonly used diagnostic tool for evaluating GERD, where a flexible tube with a camera is inserted through the mouth to visualize the esophagus and stomach [5,6]. The second technique is ambulatory pH monitoring, which measures how often and for how long stomach acid enters the esophagus over a 24 h period by inserting a small probe into the esophagus to continuously monitor pH levels [7]. The third technique is the esophageal manometry, a test that measures the pressure and function of the esophagus muscle helping to evaluate motility disorders [8,9,10]. The fourth technique is bravo pH monitoring, which involves attaching a small wireless capsule to the esophageal lining during an endoscopy. This capsule continuously measures pH levels over several days and transmits the data wirelessly to a receiver worn by the patient [11]. Finally, esophageal impedance testing differs from pH monitoring, as it can detect both acid and non-acid reflux. It is performed by inserting a probe through the nose into the lower part of the esophagus to measure changes in the complex impedance caused by liquids [8,12]. A common characteristic of all GERD measurement techniques is their discomfort for the patient, as these procedures often require prolonged monitoring over several hours or days, significantly disrupting daily activities and preventing a normal lifestyle during this period. An alternative proposed in this study is the use of radiofrequency tomography measurements, which enable the noninvasive detection of GERD, allowing patients to undergo assessment without discomfort and maintain their normal daily activities.

Spectrophotometry, defined by Maxwell’s equations, can be expressed in terms of permittivity as a complex number. The real part, or dielectric constant (ε’), is related to a tissue’s ability to store electric energy, while the imaginary part, or dielectric loss factor (ε”), is associated with the dissipation of electric energy [13]. In the RF and microwave (MW) ranges, different dispersions occur across the electromagnetic spectrum, with α, β, and γ dispersions being the most relevant [14].

The α dispersion, occurs at frequencies from a few Hz to a few kHz and is related with the phenomenon of the charges with mobility, either dissolved or suspended in a liquid phase. β dispersion typically occurs between tens of kHz and tens of MHz and involves mechanisms related to the orientation of fixed charges on solid surfaces, such as proteins and carbohydrates [15,16,17]. At higher frequencies, interactions are dominated by surface tension charges, a phenomenon known as the Maxwell–Wagner effect [18]. Finally, in the MW range, the γ dispersion appears, arising mainly from the orientation in the sense of the electric field polarization and the induction of the dipolar molecule [19,20].

Previous studies have demonstrated the usefulness of dielectric properties as an online control system in various applications, such as assessing meat quality [21,22,23], monitoring the salting process in meat or cheese [21,24], and detecting water content in biological tissues [25,26,27].

The objective of this study is to develop a radiofrequency spectrophotometry sensor capable of non-invasively detecting fluid movement and ionicity in the esophagus through the thoracic plexus, with the future aim of identifying gastroesophageal reflux disease (GERD) episodes.

## 2. Materials and Methods

### 2.1. Population

In this study, a sample of 49 individuals was evaluated, with participants stratified by age and gender to ensure statistical representativeness. The population was divided into groups based on specific age ranges and gender, allowing for a comparative analysis of variables across different demographic categories. This stratification enabled the assessment of potential variations in the measured parameters that could be associated with age- and gender-specific physiological differences. Statistical techniques were applied to assess the significance of these differences across the distinct demographic groups, providing a robust framework for interpreting the findings.

### 2.2. Characterization of Each Individual

Participants were asked whether they had any electronic devices implanted in their bodies prior to the study. This information was collected to ensure that any implanted devices would not interfere with the measurements and to maintain patient safety throughout the testing procedures. Additionally, participants were asked about cardiovascular conditions, following the ethics committee’s recommendation. Moreover, participants were instructed to refrain from consuming any food or beverages, including water, for at least two hours prior to testing to minimize potential interference with data interpretation.

Participants were asked to report their smoking status and to disclose any known digestive issues, including a history or current diagnosis of reflux, gastritis, or chronic stomach pain. These questions aimed to identify potential confounding variables related to digestive health that might impact the study’s outcomes. This information allowed for a more comprehensive understanding of each participant’s gastrointestinal health profile, facilitating a thorough analysis of factors that might influence the primary measurements.

Before testing, each participant’s height was measured, and body composition metrics were assessed using a bioimpedance scale (BF511 OMRON, Kyoto, Japan). This device provided measurements of body weight, body fat percentage, protein content, and visceral fat levels. These parameters were collected to create a detailed profile of each participant’s physical characteristics, enabling a more in-depth analysis of potential correlations between body composition and study outcomes.

### 2.3. Impedance Analyser to Obtain Permittivity Measurements

**Figure 1:**
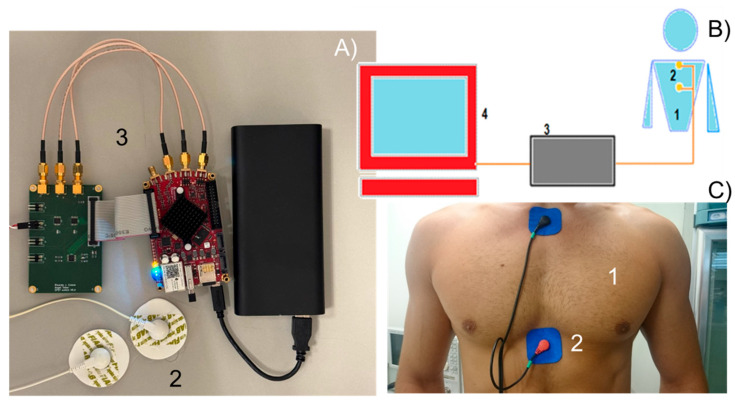
Sensor developed to measure dielectric properties across the thoracic plexus and reach the esophagus. (**A**) represents the adhesive ECG electrodes (**2**) and the analyzer prototype (**3**); (**B**) represents a scheme of the measurer system, where 1 is the thoracic plexus, 2 is the electrodes, 3 is the analyzer, and 4 is the computer; and (**C**) represents a picture of the thoracic plexus (**1**) with the adhesive ECG electrodes (**2**) applicated in the measured disposition.

Impedance measurements were conducted using a prototype built with commercial hardware, featuring an on-chip system based on a programmable gate array. Specifically, the Redpitaya Stemlab 125-14 board (Solkan, Slovenia) was employed. This board is based on a field-programmable gate array (FPGA) SoC Zynq 7010 from Xilinx, which has a programmable logic zone of 28,000 logic cells together with which a dual-core Cortex-A9 microprocessor is integrated. The FPGA is connected to 512 MB of DDR3 RAM, has 2 analog-to-digital converters at 125 MS/s and 14 bits of resolution, 2 devices to convert binary data into current signals at 125 MS/s and 14 bits of resolution (Figure 1A3). The inputs and outputs have a bandwidth of 60 MHz. Adhesive ECG electrodes (Figure 1A2,C2) were used as sensors to interface the impedance analyzer with the test subject. Figure 1B illustrates the measurement system used in this study, combining a schematic diagram and a photograph that identifies its key components.

### 2.4. Procedure

To ensure consistency across all measurements, specific brands of water and juice were selected under controlled conditions. Commercial brand water, packaged in 2 L containers, was used as the standard for water measurements. For juice, which is highly perishable, 1 L cartons of UHT-treated orange juice from the same brand were selected. This approach minimized variability and ensured reproducible conditions in each testing session. Additionally, a 1% (*w*/*w*) sodium chloride solution was prepared using the same brand of water employed in the pure water measurements. This standardization of the water source ensured consistency across solution preparations and minimized potential variations in electrolyte content due to differences in water composition.

With electrodes positioned over the sternum and the upper stomach region (Figure 1) and connected to the base plate, the inter-electrode distance was measured for each participant. Four measurements were recorded per individual: Measurement 0, taken in the absence of liquid; Measurement 1, while drinking water; Measurement 2, while drinking juice; and Measurement 3, while drinking a 1% saline solution. To ensure consistency, participants were instructed to consume the full glass of each liquid, signaling when they were halfway through. This timing allowed measurements to be taken when the esophagus was fully occupied by the liquid, ensuring accurate impedance data during peak liquid transit through the esophagus.

### 2.5. Statistical Analysis

The statistical analysis was carried out with the Statgraphics Centurion XIX Software (Statgraphics, The Plains, VA, USA). One-Way ANOVA analyses were performed in order to find statistically significant differences between the studied parameters. The logistic Traffano–Schiffo model [14] was fitted by using nonlinear regression. Finally, the predictive algorithm was developed using the multiple regression tool.

## 3. Results

The participants’ measurements provided the following demographic and physical characteristics, summarized in Table 1. This table presents the statistical distribution of parameters relevant to impedance measurement analysis. It includes mean values, with minimum and maximum values indicated in parentheses, and classifies subjects based on characteristics that may influence in the measurements.

The classification presented in Table 1 is based on the guidelines provided by Gallagher et al. [28], which indicate that body fat percentage cutoffs for overweight status vary slightly by age and sex. Men are typically classified as overweight with body fat levels ranging from 25 to 29.9%, while women are classified as overweight with levels between 30 and 34.9%. Normal weight is defined as a body fat percentage between 8.1% and 16% in men and between 15.1% and 20.9% in women. These thresholds are consistent with findings from other studies [29]. The mass fraction of visceral fat is increasingly used to assess obesity-related health risks, with specific thresholds indicating elevated risk levels for both men and women. A visceral fat mass fraction exceeding 10–20% of total body fat is associated with increased risks of metabolic syndrome, cardiovascular disease, and type 2 diabetes, with sex-specific thresholds. In this context, men with more than 15% visceral fat and women with more than 10% are generally considered to be at higher risk for these conditions due to the distribution and metabolic activity of visceral fat [30].

### 3.1. Participant Health and Lifestyle Information

Participants provided responses on lifestyle factors and digestive health through an anonymous survey. Data on smoking status, digestive issues, and incidence of reflux, gastritis, or chronic stomach pain were recorded. Of the 49 participants, 6 (2 men and 4 women) identified as smokers, and 43 as non-smokers. A total of 7 individuals reported digestive issues (3 men and 4 woman), while 42 did not. Additionally, 6 participants indicated a history of reflux, gastritis, or chronic stomach pain (2 men and 4 woman), while 43 reported no such symptoms.

**Figure 2:**
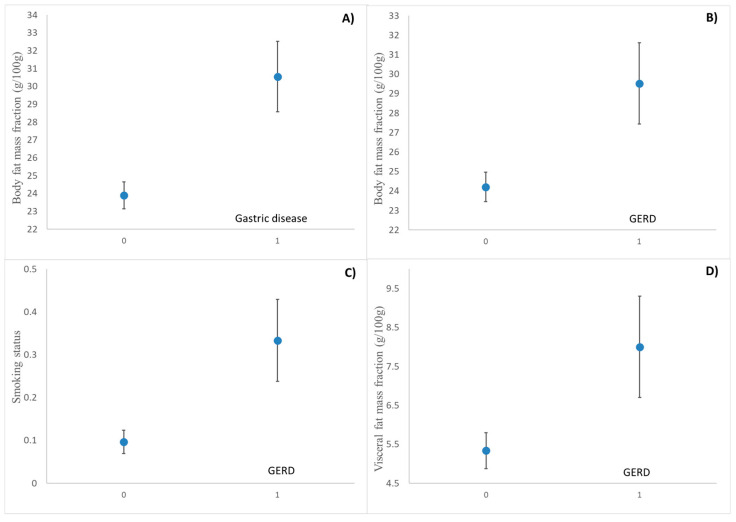
Effect of health and lifestyle on the incidence of gastric problems and, in particular, the incidence of reflux in LSD intervals. (**A**) The the effect of body fat fraction on the incidence of general gastric problems. (**B**) The effect of body fat fraction on the incidence of gastroesophageal reflux. (**C**) Smoking status versus the incidence of gastroesophageal reflux. (**D**) The visceral fat content versus the incidence of gastroesophageal reflux.

Three dummy variables were created to categorize subjects based on their smoking status, reflux presence, and incidence of gastric issues. Each variable was coded as a binary indicator, where a value of 0 represents the non-smoker study participants and the study participants without gastric disease incidences (no gastroesophageal reflux or any gastric incidence), and a value of 1 represents the smoker study participants and the study participants with gastric disease incidences (diagnosed with gastroesophageal reflux disease, GERD, or diagnosed with any other gastric disease). This binary classification enables straightforward inclusion in regression models, facilitating the analysis of associations between these factors and health outcomes. Figure 2 illustrates the effect of various health factors, such as body fat mass fraction, visceral fat mass fraction, and smoking activity, on digestive issues.

As shown in Figure 2A, the mass fraction of body fat has a very significant effect on gastric incidents, as reflected in Figure 2B, which also affects gastroesophageal reflux [31]. Furthermore, as can be seen in Figure 2D, the effect of visceral fat content on the incidence of gastroesophageal reflux is also very significant [32], possibly because the total fat content is directly related to the visceral fat content. Finally, in Figure 2C, a highly significant association is observed between smoking habits and the incidence of gastroesophageal reflux. This relationship indicates that smoking may be a critical factor influencing the frequency or severity of reflux. These effects have been reported by other authors [33,34].

### 3.2. Sensor Design and Adaptation to Achieve the Required Depth

The developed sensor comprises two superconducting poles, each with an adhesive backing, which are strategically placed on the subject’s body to capture data. One pole is placed on the trachea, while the other is attached at the xiphoid process, near the end of the sternum, precisely aligned with the cardia. This dual-pole configuration allows for sensitive measurements across the target regions. The sensor is connected to an adapted commercial impedance analyzer, as previously described.

**Figure 3:**
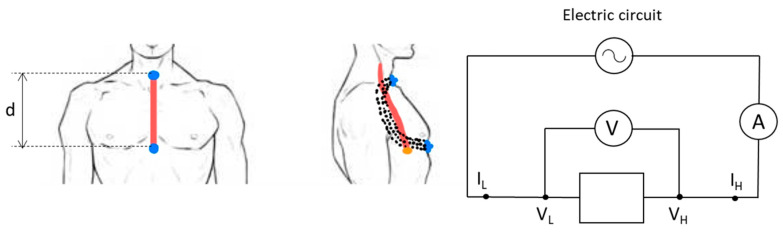
Positioning of the two poles (blue) to allow the signal to penetrate the esophagus (red) and to measure the cardiac valve (orange). The electric field lines are in black. The measurement “d” represents the distance between the poles. On the right, the measurement circuit is shown, designed to determine the intensity and the voltage difference between the poles and to be able to calculate the complex permittivity.

Figure 3 illustrates the sensor placement on the subject’s body during the analysis. In this figure, it is possible to observe how the electric field lines reach the entire esophagus, including the cardia. The signal obtained by the prototype analyzer is the impedance ($\bar{Z}$). Taking into account that the impedance is a vector and can be expressed as a complex number as $\bar{Z}=R+jX$, where the real part of the impedance is the resistance R and the imaginary part is the reactance X, it is possible to estimate ε′, ε″ by using R and X parameters, as follows (Equations (1)–(3)):

(1) $$\varepsilon^{\prime }(f)=\frac{-X}{\left(R^{2}+X^{2}\right)}\frac{1}{2\pi {fC}_{0}}$$

(2) $$\varepsilon^{\prime \prime }(f)=\frac{R}{\left(R^{2}+X^{2}\right)}\frac{1}{2\pi ƒC_{0}}$$

(3) $$C_{0}=\frac{\varepsilon_{0}S}{d}$$

where ƒ is the frequency (Hz), $C_{0}$ is the capacitance in the vacuum (F), S is the surface of the electrodes (m^2^), $\varepsilon_{0}$ is the vacuum permittivity (F/m), and d is the separation between the electrodes with differential tension (V_H_-V_L_) (m) (see detail in Figure 3).

The measurement technique employed in this study is based on the circuit illustrated in Figure 3. It involves applying a low-amplitude excitation signal to the sample under test in order to measure the resulting voltage and current responses. The excitation signal is digitally generated by a digital-to-analog converter (DAC), which produces a voltage signal. To inject this signal as current into the sample, a controlled current source is used.

The system is designed to measure both the voltage drop and the current flow across the sample during excitation. This is achieved through a signal conditioning stage followed by two analog-to-digital converters (ADCs) that capture signals proportional to the voltage and current, respectively. By applying appropriate signal processing algorithms—in conjunction with prior knowledge of the excitation signal and the recorded measurements—the impedance spectrum of the sample can be calculated. Furthermore, by incorporating information about the sensor geometry and configuration, the dielectric permittivity of the material can also be estimated.

The data processing pipeline includes signal interpretation, feature extraction, and condition assessment of the sample. Processing may be conducted in real time on the acquisition hardware, enabling rapid analysis, adaptive measurement protocols based on intermediate results, and fast decision-making. Alternatively, data can be transmitted to an external PC-based system for remote processing and more complex computational tasks.

Moreover, it is possible to estimate the ionic conductivity using the loss factor, frequency, and vacuum permittivity, as follows (Equation (4)):

(4) $$\sigma =\varepsilon_{0}\varepsilon^{\prime \prime }2\pi f$$

where σ is the conductivity, expressed in S·m^−1^.

**Figure 4:**
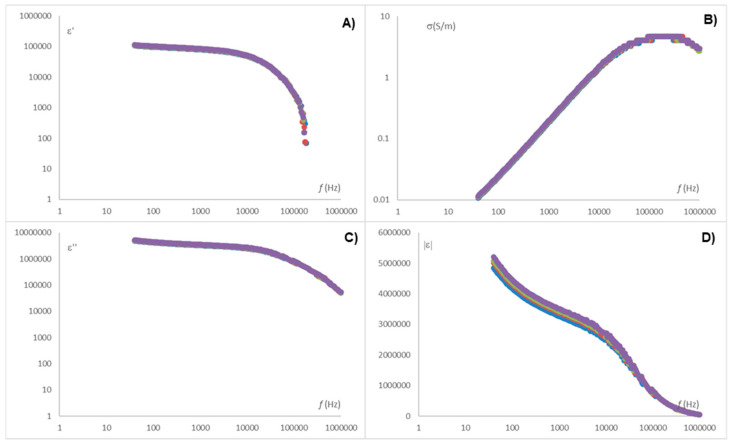
Permittivity spectra in radiofrequency range for Subject 39 (woman, 39 years old, 161 cm of height and 69.8 kg of weight): (**A**) dielectric constant, (**B**) loss factor, (**C**) ionic conductivity (S/m), (**D**) modulus of permittivity. (●) The spectrum measured with an empty esophagus, (●) when the esophagus was filled with water, (●) when the esophagus was filled with orange juice, and (●) when it was filled with 1% brine.

As shown in Figure 4, the dielectric constant spectrum exhibits two distinct regions at low and high frequencies, corresponding to the alpha and beta relaxations, respectively. Each relaxation represents a different interaction phenomenon between the electric field and the chemical species present. Therefore, for accurate interpretation of the results, it is essential to obtain the parameters for both the alpha and beta relaxations. One approach to determining these relaxation parameters is the use of the Traffano–Schiffo model (2017) [14], based on Gompertz logistic models, which are widely applied in biological systems [22,27]. Equation (5) presents the Traffano–Schiffo model:

(5) $$l\varepsilon^{\prime }(\omega )=l{\varepsilon^{\prime }}_{\infty }+\sum_{n=1}^{3}\frac{\Delta l^{\prime }\varepsilon_{n}}{1+e^{\left((l\omega^{2}-l{\varpi_{\tau }}^{2})*\alpha_{n}\right)}}$$

where Ɩε′ represents the decimal logarithm of the dielectric constant; ${Ɩ\varepsilon^{\prime }}_{\infty }$ the logarithm of the dielectric constant at high frequencies; Ɩω represents the decimal logarithm of the angular velocity (obtained from the frequency); ${\Delta Ɩ\varepsilon^{\prime }}_{n}$ (${\Delta Ɩ\varepsilon^{\prime }}_{n}$= ${\log\varepsilon^{\prime }}_{n}-{\log\varepsilon^{\prime }}_{n-1})$ the magnitude of the dispersion; ${Ɩ\omega }_{t}$ the logarithm of the angular velocity at relaxation time for each dispersion n; and $\alpha_{n}$ are the dispersion slopes.

From the Gompertz parameters, applied only for alfa and beta dispersion, it is possible to determine the relaxation frequencies and dielectric constants of each relaxation (Equations (6)–(8)).

(6) $${\varepsilon^{\prime }}_{\alpha }=10^{\left(l{\varepsilon^{\prime }}_{\infty }+\Delta l{\varepsilon^{\prime }}_{\beta }+\frac{\Delta l{\varepsilon^{\prime }}_{\alpha }}{2}\right)}$$

(7) $${\varepsilon^{\prime }}_{\beta }=10^{\left(l{\varepsilon^{\prime }}_{\infty }+\frac{\Delta l{\varepsilon^{\prime }}_{\beta }}{2}\right)}$$

(8) $$f_{i}=10^{\frac{l\varpi_{\tau i}}{2\cdot \pi }}$$

With i being subscript for Equation (8) in each dispersion (α, β).

### 3.3. Statistical Analysis on Relaxation Values

**Figure 5:**
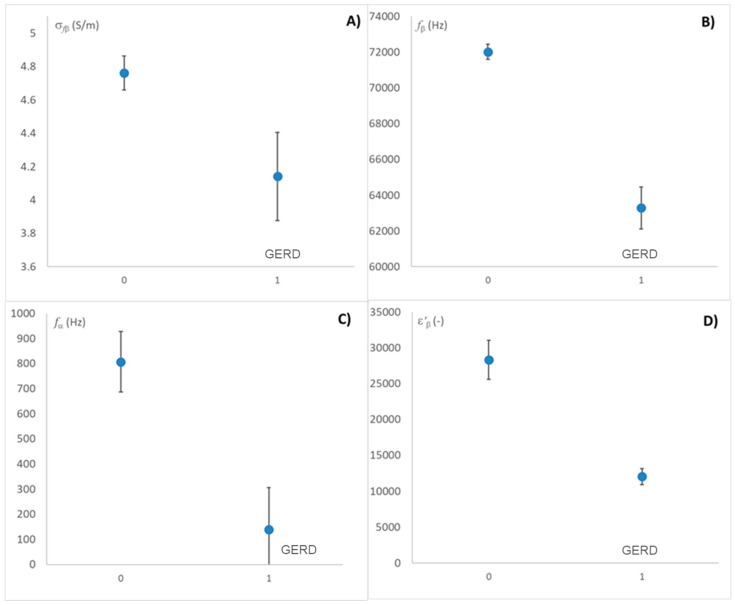
The effect of suffering from reflux incidence on relaxation values. (**A**) Ionic conductivity at β relaxation (S/m). (**B**) The frequency in β relaxation. (**C**) The frequency in α relaxation. (**D**) The dielectric constant in β relaxation. All graphs are represented with LSD intervals.

The effect of digestive problems or reflux on relaxation values was analyzed first to determine whether individuals experiencing reflux or digestive issues exhibit a different response compared to the rest of the subjects. As in Figure 2, in Figure 5, a binary encoding was used, where ‘1’ denotes the presence of the disease and ‘0’ its absence.

As shown in Figure 5, the measurements (including those of all liquid media) influence certain relaxations, primarily the beta relaxations. Specifically, individuals affected by reflux exhibit beta differentiation, which may be due to damage to the esophageal muscular tissue. This is because beta scattering represents photon interactions with fixed charges in macromolecules such as proteins. Therefore, the beta scattering spectrum can be a good discriminator of the appearance of damage caused by the action of reflux on the esophageal walls. This phenomenon is observed in Figure 5A,B,D.

However, Figure 5C shows the difference in the alpha relaxation frequency, that is, when taking the following liquids, there is an effect of other ionic chemical species from the esophagus itself that affects the measurement and that will make it difficult to detect the release of chloride ions from the stomach.

**Figure 6:**
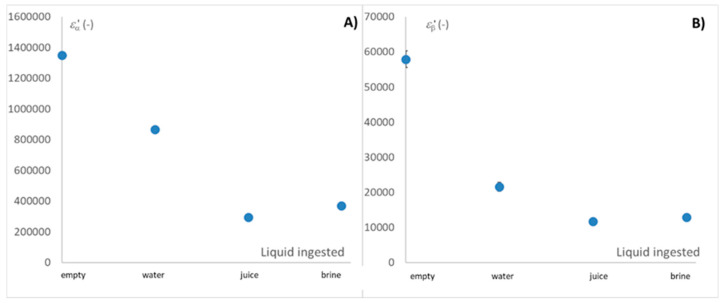
Dielectric constant in relaxation frequencies related to the ingested liquid flowing throughout the esophagus: (**A**) α relaxation and (**B**) β relaxation. Represented with LSD intervals.

A comparison of the measurements for the intake of each of the liquid media is shown in Figure 6.

As observed in Figure 6, the dielectric constant in both alpha and beta relaxations shows significant differences for each liquid, with the exception of juice and brine. In the case of alpha dispersion, this could be attributed to the higher ionic strength of both juice and brine. Furthermore, as shown in Figure 5C, interference is associated with the degradation of the esophagus and the release of native electrolytes in individuals suffering from reflux. In the case of beta dispersion, the effect shown in Figure 5D on the incidence of reflux on esophageal tissue, together with Figure 6B, which shows the effect of the intake of different liquids, suggests that the degradation of esophageal muscle tissue may possibly cause electrolyte measurements in a medium degraded by reflux to minimize the difference between juice and brine.

**Figure 7:**
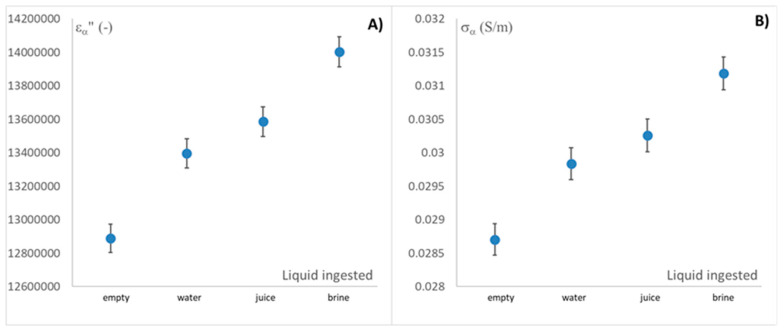
Dielectric properties at α dispersion related to the ingested liquid flowing throughout the esophagus: (**A**) loss factor and (**B**) ionic conductivity (S/m). Represented with LSD intervals.

Since alpha dispersion directly interacts with the ionic strength of the medium, and the ionic strength of the different liquid media is arranged from lowest to highest, in the following order: water, juice, and brine. The different dielectric properties of this relaxation should be perfectly arranged. However, as seen in Figure 6A, this is not the case for liquids with higher ionic strengths, so the nature of the charges may influence the distribution of the dielectric constant values. Furthermore, since they are strongly ionic systems, alpha and beta dispersion may be distorted in the loss factor by ionic conductivity, which can mask the loss spectrum when ionicity is very high. For this reason, Figure 7 shows the different average values of the loss factor and ionic conductivity in alpha dispersion, represented as LSD intervals.

As can be seen in Figure 7A,B, the alpha loss factor follows the same trend as the ionic conductivity, which determines that the loss factor is completely masked by the conductivity.

Furthermore, this figure shows that, importantly, the measurement of ionic conductivity in alpha scattering is able to perfectly differentiate the intake of each liquid and distinguish it from the empty esophagus. This opens the door to detecting gastroesophageal reflux with the proposed measurement system.

## 4. Discussion

The results of this study demonstrate that radiofrequency spectrophotometry is a viable non-invasive technique for detecting gastroesophageal reflux disease (GERD) by measuring esophageal liquid flow and ionicity. The ability of the developed sensor to distinguish between different liquid media suggests a high sensitivity to changes in esophageal content, making it a promising alternative to traditional diagnostic methods more invasives. These findings align with previous research on the use of dielectric properties for biomedical applications, where variations in permittivity and ionic conductivity have been effectively used to characterize physiological changes in different tissues [16,35]. A key outcome of this study is the observation that beta dispersion relaxation is particularly affected in individuals with GERD, which may indicate esophageal tissue damage due to prolonged acid exposure. The potential of beta dispersion as a biomarker for GERD-induced tissue changes further supports the use of dielectric-based techniques in gastroenterology. Furthermore, the ability of the developed sensor to differentiate alpha dispersion properties in various ingested liquids suggests that ionic conductivity measurements can effectively track reflux events in real-time. The present study builds upon these findings by demonstrating that alpha dispersion-based measurements are particularly relevant in GERD diagnostics, as they reflect both the presence of refluxed liquid and the esophagus’ physiological response to different ionic environments.

The study also confirms strong correlations between lifestyle factors and GERD incidence, particularly regarding visceral fat content and smoking habits. These results are in agreement with Koo et al. [32] and Valezi et al. [31], who found that obesity and excess visceral fat increase intra-abdominal pressure, leading to weakened lower esophageal sphincter function and a higher prevalence of GERD symptoms. Similarly, Ness-Jensen and Lagergren [34] reported a direct association between smoking and GERD severity, reinforcing the present study’s findings that smoking significantly contributes to reflux events. The consistency of these results with prior studies highlights the need to consider patient-specific physiological characteristics in GERD diagnostics and treatment strategies.

Recent advances in Raman spectroscopy have demonstrated promising potential for the diagnosis of esophageal diseases, mostly in cancer diseases. For instance, Hiremath et al. (2019) [36] reviewed the clinical translation of Raman spectroscopy for in vivo biochemical characterization of esophageal tissue, highlighting its utility during endoscopic procedures. More recently, Fan et al. (2025) [37] introduced dual-wavelength Raman spectroscopy, which further enhanced spectral resolution and improved diagnostic sensitivity in distinguishing between pathological and normal tissue states. While these approaches rely on optical interactions and typically require direct visualization of the esophageal mucosa, our study presents a fundamentally different and fully non-invasive diagnostic alternative. Furthermore, the small size and weight of the device allows patient measurements to be taken for 24–48 h without causing discomfort, allowing the patient to lead a normal life. Specifically, in this study, a radiofrequency spectrophotometry sensor was developed, capable of detecting esophageal liquid flow and ionicity through the thoracic plexus, without the need for invasive probes or endoscopic access. By identifying beta dispersion relaxation as a biomarker of tissue damage and alpha dispersion conductivity as an indicator of reflux episodes, our sensor enables real-time, ambulatory monitoring of GERD. This approach complements Raman-based techniques by focusing on functional and dielectric properties of the esophagus, opening new avenues for continuous, patient-friendly GERD diagnosis, and long-term management.

Overall, this study reinforces the potential of radiofrequency spectrophotometry as a non-invasive, real-time GERD diagnostic tool. The ability to track esophageal liquid movement and tissue response with high sensitivity represents a significant advancement over current invasive techniques. By minimizing patient discomfort while maintaining diagnostic accuracy, this approach could be further optimized for continuous monitoring applications, paving the way for a new era of GERD detection and management.

## 5. Conclusions

This study successfully developed and evaluated a radiofrequency spectrophotometry sensor capable of non-invasively detecting esophageal liquid flow and ionicity, providing a novel approach for diagnosing gastroesophageal reflux disease (GERD). The sensor demonstrated its ability to differentiate between various liquid media, offering a highly sensitive method for detecting reflux events without requiring invasive procedures.

The results indicate that dielectric properties in the radiofrequency range can effectively characterize esophageal content, with significant variations observed in alpha and beta dispersions depending on the liquid type. Notably, the beta dispersion relaxation was found to be particularly relevant in individuals with reflux, suggesting its potential as a biomarker for esophageal tissue damage. Additionally, the measurement of ionic conductivity in alpha dispersion successfully distinguished between different ingested liquids, opening up new possibilities for detecting GERD episodes in real time.

Statistical analysis confirmed the relationship between body composition parameters, lifestyle factors (such as smoking), and the incidence of reflux, reinforcing the importance of considering patient-specific physiological characteristics in GERD diagnostics. The observed correlations suggest that visceral fat content and smoking habits significantly influence reflux frequency and severity, supporting previous findings in gastroenterological research.

Overall, this study provides a groundbreaking alternative to traditional GERD diagnostic methods, offering a non-invasive, real-time monitoring tool that minimizes patient discomfort while maintaining high diagnostic accuracy. Future research should focus on optimizing the sensor’s performance, validating it in larger clinical trials, and exploring its potential applications in continuous GERD monitoring outside of clinical settings.

## Figures and Tables

**Table 1:** Population summary table: characteristics listed include the measured parameter and respective units, with averages, minimums, and maximums presented.

Population Parameters								Mean	
**Age (years)**								***n* = 49**	
18 < x ≤ 30		30 < x ≤ 50		50 < x ≤ 70				M	W
M	W	M	W	M	W			28.1	35.4
17	12	5	11	1	3			[18–53]	[20–56]
**Height (cm)**									
≥160		160 < x ≤ 170		170 < x ≤ 185		185 < x			
M	W	M	W	M	W	M	W	178.4	163.0
0	8	3	16	15	2	5	0	[164–190]	[151–176]
**Weight (kg)**									
≥50		50 < x ≤ 65		65 < x ≤ 90		90 < x			
M	W	M	W	M	W	M	W	80.3	58.2
0	6	3	11	14	8	6	1	[54–119]	[43–85]
**Body Fat mass fraction (g/100 g)**									
≥18.5		18.5 < x ≤ 26.9		26.9 < x ≤ 40		40 < x			
M	W	M	W	M	W	M	W	19.5	26.0
9	1	10	13	4	8	0	4	[0.7–38]	[18–45]
**Visceral Fat mass fraction (g/100 g)**									
≥3		3 < x ≤ 5		5 < x ≤ 9		9 < x < 24			
M	W	M	W	M	W	M	W	7.3	4.1
2	6	5	12	10	7	6	1	[2–23]	[2–9]
**Skeletal protein mass fraction (g/100 g)**									
≥25		25 < x ≤ 30		30 < x ≤ 40		50 < x			
M	W	M	W	M	W	M	W	40.1	29.3
0	3	1	13	11	10	11	0	[28–47]	[23–34]
**Body mass index (BMI)**									
≥20		20 < x ≤ 22		22 < x ≤ 25		25 < x < 40			
M	W	M	W	M	W	M	W	25.3	21.8
1	12	3	6	12	1	7	7	[19–37]	[17–34]

## Data Availability

The raw data are available to the entire scientific community in the Zenodo database with the following DOI: 10.5281/zenodo.15050838.

## Abbreviations

The following abbreviations are used in this manuscript:

GERD	gastroesophageal reflux disease
FPGA	field-programmable gate array
UHT	ultra-high temperature
LES	lower esophageal sphincter
